# Incorporating Ecosystem Services in the Assessment of Water Framework Directive Programmes of Measures

**DOI:** 10.1007/s00267-021-01478-7

**Published:** 2021-05-12

**Authors:** Ioannis Souliotis, Nikolaos Voulvoulis

**Affiliations:** Centre for Environmental Policy, Imperial College London, London, UK

**Keywords:** Water Framework Directive, Programme of Measures, Effectiveness analysis, Ecosystem services

## Abstract

The EU Water Framework Directive requires the development of management responses aimed towards improving water quality as a result of improving ecosystem health (system state). Ecosystems have potential to supply a range of services that are of fundamental importance to human well-being, health, livelihoods and survival, and their capacity to supply these services depends on the ecosystem condition (its structure and processes). According to the WFD, Programmes of Measures should be developed to improve overall water status by reducing anthropogenic catchment pressures to levels compatible with the achievement of the ecological objectives of the directive, and when designed and implemented properly should improve the ecological condition of aquatic ecosystems that the delivery of ecosystem services depends on. Monitoring and evaluation of implemented measures are crucial for assessing their effectiveness and creating the agenda for consecutive planning cycles. Considering the challenges of achieving water status improvements, and the difficulties of communicating these to the wider public, we develop a framework for the evaluation of measures cost-effectiveness that considers ecosystem services as the benefits from the reduction of pressures on water bodies. We demonstrate its application through a case study and discuss its potential to facilitate the economic analysis required by the directive, and that most European water authorities had problems with. Findings demonstrate the potential of the methodology to effectively incorporate ecosystem services in the assessment of costs and benefits of proposed actions, as well as its potential to engage stakeholders.

## Introduction

The EU Water Framework Directive (WFD) recognizes that water ecosystems do not constitute stand-alone structures, but are embedded within a wider socio-ecological system and proposes River Basin Management Plans (RBMPs) as the means of achieving the protection, improvement and sustainable use of freshwater systems across Europe. At the core of RBMPs, Programmes of Measures (PoMs) aim to protect the environment and improve the overall status of the system (Voulvoulis et al. 2017; Vugteveen et al. 2006). PoMs constitute tailored actions implemented by the managing authorities to reduce catchment pressures to levels that are compatible with the achievement of the ecological objectives (i.e. good status of water bodies) introduced by the WFD (Giakoumis and Voulvoulis 2018b). In developing PoMs, the WFD (Art. 11, par.1) requires Member States to utilize the information gathered in fulfilling the provisions of earlier articles (e.g. Article 5 on the characterisation of the river basin district) and the gap analysis between the current status and the reference conditions. Monitoring and evaluation of implemented measures are crucial for assessing their effectiveness and creating the agenda for the consecutive planning cycle (Europen Commission 2012).

However, from the submitted RBMPs, as well as published European Commission reports (European Commission 2019), it is clear that significant gaps exist in the assessment of PoMs. The 4th Implementation Report (European Commission 2015) published in 2015, raised concerns about the economic analysis and the link between pressures and PoMs in providing justification for their selection by most Member States. Cost-effectiveness analysis (CEA) had been suggested by the WATECO group (European Commission 2003) and had been adopted by most states (Martin-Ortega 2012) as part of the WFD implementation process. However, its application, as reported in the 1st cycle of the RBMPs varied among countries, with only 8 (Germany, France, Lithuania, Luxembourg, Latvia, Portugal, Romania and United Kingdom) out of the 23 countries including this type of analysis when designing measures (European Commission 2015). Even in these countries, it was not treated consistently across river basins, with some not mentioning it at all or including a general description (WRc 2015). Differences in the depth of analysis among Member States were also confirmed by the 5th Implementation Report, published in February 2019. More specifically, ambiguity was observed about what costs should be included in the assessment of PoMs, with only one third of the total number of assessed Member States providing full information (European Commission 2019). Significant gaps remain in achieving more harmonised approaches to estimate and integrate environmental and resource costs, while it is acknowledged that the economic underpinning of PoMs would greatly facilitate water-related decisions and investments (Gómez-limón and Martin-Ortega 2013). What often seems to lack in environmental management decisions is the connection between pressures and ecosystem functions (Schröter et al. 2019), which negatively influences economic decisions.

Ecosystems have the potential to supply a range of services that are of fundamental importance to human well-being, health, livelihoods and survival (Costanza et al. 1997; MEA 2005; TEEB 2010), and these services can be described as the benefits that people obtain from ecosystems (MEA 2005). Recent publications have defined ecosystem services as contributions of ecosystem structure and function (in combination with other inputs) to human well-being (Burkhard et al. 2012; Burkhard and Maes 2017). The concept of ecosystem services was developed in the 1990s as a way to improve the effectiveness of biodiversity-protection policies (Fisher et al. 2008). Conceptually, it considers the links of biodiversity and ecosystems with socio-economics systems (Boulton et al. 2016). With a global initiative on the economics of ecosystems and biodiversity, which started in 2007, setting a framework for valuing ecosystem services (Bourguignon 2015), their application to improve economic analysis and contribute to several aspects of the WFD implementation has been acknowledged (Grizzetti et al. 2016; Vlachopoulou et al. 2014). Their application has been shown to allow for a more systematic way to effectively prioritise significant pressures and therefore select appropriate PoMs for the WFD (Giakoumis and Voulvouli 2018a), and has been suggested for the assessment of policies (Nyborg 2014). However, their potential to improve the economic underpinning of PoMs and evaluate their effectiveness in economic terms has been underexplored. Here therefore, we develop a framework for the evaluation of the effectiveness of PoMs that considers ecosystem services, as the benefits from improvements in overall water status classifications. We demonstrate its application through a case study where we evaluate PoMS by comparing expected costs and benefits associated with changes in the delivery of ecosystem services due to their implementation and discuss its potential to facilitate the economic analysis required by the directive. In our methodology, we accommodate stakeholders’ perceptions and public preferences, as required by the WFD (Article 14) for the design and selection of PoMs (Perni et al. 2020) and the overall implementation of the directive (Waylen et al. 2019).

## Incorporating Ecosystem Services in the Economic Analysis Concerning PoMs

Economic principles and instruments are at the core of the WFD and vital for its success, as several of its articles require Member States to undertake economic analysis. Article 4 requires economic appraisal of disproportionate costs to assess the need of exemptions; Article 5 sets the deadline for the preparation of economic analysis of water uses; Article 9 requires the assessment of the level of recovery of costs for water services and Article 11 and Annex III state that the cost-effectiveness of PoMs should be assessed.

Overall, the issue of effectiveness of adopted policy is central to the WFD and directly related to economic principles. However, as at the time when the WFD was introduced, only a few Member States had experience with using economic approaches in environmental management, the initial reports of the Member States were not able to fulfil the economic analysis requirements for assessing effectiveness (Kanakoudis and Tsitsifli 2010). To assist Member States with these aspects of the WFD, the European Commission published several guidance documents. The first of its kind was the WATECO document (European Commission 2003) that was developed under the Common Implementation Strategy (CIS) process, which aimed to foster the harmonisation of economic knowledge in the field of water economics throughout Member States. In 2006, a CEA document (CEA Drafting Group 2006) was drafted aiming to prepare the Member States to undertake a more integrated approach to decision-making. After that, a few more studies were developed to provide a standard approach to assessing the effectiveness of PoMs and provide guidance on how to account for their costs and benefits (European Commission 2010; Nocker et al. 2007).

Generally, though the WFD does not explicitly require the implementation of specific methods, the supporting documents have focused on two approaches, namely CEA and cost–benefit analysis (CBA) for the evaluation of PoMs. One of the main differences between the two methods is that CEA compares costs and physical benefits, whereas CBA compares monetarily valued social, environmental and economic costs and benefits^1^. Over the years, the European Commission has been supporting cost–benefit assessments, in relation to the costs of PoMs, the benefits of reaching good water status as well as the costs of not achieving the WFD objectives among others (Boeuf et al. 2016). In addition, England, Scotland, France (Seine, Normandy), the Netherlands and Denmark have shown a strong preference for CBA instead of CEA. However, the lack of a unified framework has often resulted in confusion about what costs and benefits should be accounted for in relevant analyses (Greenhalgh et al. 2017), resulting in weak assessments according to the 5th Implementation report (European Commission 2019).

In 2009, in a CIS document (European Commission 2009) that concerned exemptions under Article 4, the Commission made the first explicit reference to ecosystem services in the context of WFD, signalling the potential added value of integrating this concept to economic analysis to improve its outputs (Bouwma et al. 2018). Ever since, supporting documents related to the economics of the WFD have been trying to provide a clear link between ecosystem services and evaluation of policies (e.g. European Commission 2016) recognizing that they constitute a concept that can accommodate qualitative, quantitative and monetary assessments (Ozdemiroglu Allan Provins Stephanie Hime et al. 2010). In addition, there are several studies that demonstrate how ecosystem services can be operationalized for the implementation of the WFD (Heink et al. 2016; Martin-Ortega 2012; Pistocchi et al. 2016). From a systems point of view, the status of a water body could be considered as an indicator of its overall health, which affects its capacity to generate ecosystem services. PoMs, by managing the pressures in a way to improve water status, affect the functioning of the aquatic system and the consequent benefits it provides to humans. Ecosystem services can be used to frame these benefits more holistically and improve the quantification of the extent to which natural resources contribute to human well-being (Hails and Ormerod 2013).

The use of ecosystem services in economic valuation exercises has been a growing trend in the academic literature in the last decades (Birol et al. 2006; Doherty et al. 2014; Eftec 2005; Liu et al. 2010; Pavanelli and Voulvoulis 2019; Voulvoulis 2015). In these studies, different WFD status class categories are defined by the quantity of ecosystem services they provide. However, these studies do not provide an actual framework on how to integrate their results into decision-making, thus they have been rarely utilized (Laurans et al. 2013). Consequently, interdisciplinary methodologies to integrate social as well as environmental benefits that give practical explanation on how they can be applied in real cases have yet to be developed.

## An Ecosystem Services Framework for Assessing the Cost-effectiveness of PoMs

**Fig. 1:**
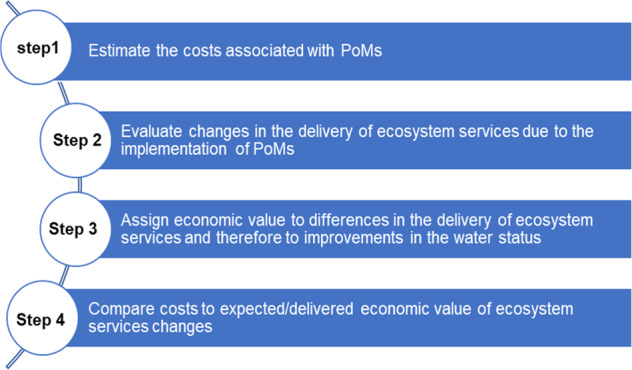
Framework for assessing effectiveness of selected or implemented PoMs

The WFD requires Member States to undertake economic analysis in order to make judgements about the best combination of measures that will achieve the Directive’s objectives. Considering the costs of the selected PoMs and their impacts on water body status through elimination of pressures expressed as changes in the delivery of ecosystem services (Grizzetti et al. 2019), here we propose evaluating PoMs effectiveness as the ratio of the value created from these changes elicited through relevant stakeholders’ preferences to the implementation costs. Taking into account the lack of harmonised approaches and that many EU Member States have not yet developed CEA methodologies; the proposed framework (Fig. 1) can help clarify the national approaches and enable their comparability.

### Step 1: Estimation of the Costs of PoMs

To assess the effectiveness of implemented PoMs, the first step includes the evaluation of all negative impacts of these measures in economic terms. The magnitude of costs may be affected by the type of measures, their duration as well as the area they target. This should be straightforward, as most competent authorities include in the RBMPs the costs estimates of the associated basic and supplementary PoMs. Such costs are capital/investment and operational costs, as well as any other negative impacts that generate welfare losses (for example, a policy intervention may increase consumer prices or decrease production output). To put that into perspective, the potential costs of measures to achieve the water body and protected areas objectives in the Anglian river basin that includes in the second RBMPs (2015–2021) are estimated to be £5050 and £4740 million (undiscounted) in the Thames river basin (Defra and Environment Agency 2015a, 2015b).

### Step 2: Evaluation of Improvements in the Provision of Ecosystem Services due to the Implementation of PoMs

**Fig. 2:**
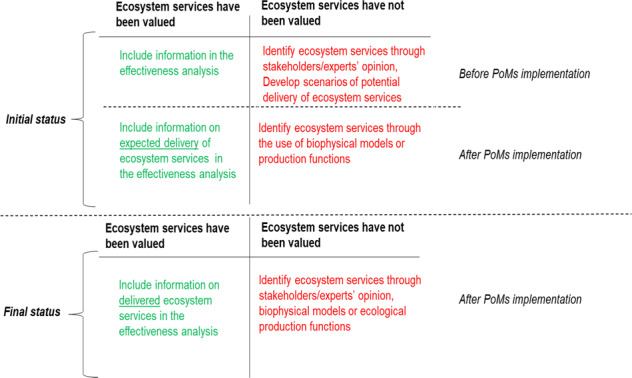
Assessment of changes in ecosystem services due to PoMs implementation

The second step aims to evaluate the effectiveness of the PoMs in improving the provision of ecosystem services through the elimination of pressures on water bodies. It involves understanding the level and type of delivery of ecosystem services before and after any policy intervention (expected or delivered). Although there is not a standardized way to identify and assess ecosystem services (Malinga et al. 2013), such information might be drawn from assessments undertaken by the managing authorities or local knowledge. The aim of this step is to evaluate the effectiveness of the PoMs as changes in the provision of ecosystems services before and after their implementation as a result of reducing pressures on water bodies. In obtaining such information, the following cases are recognized (Fig. 2).

**Fig. 3:**
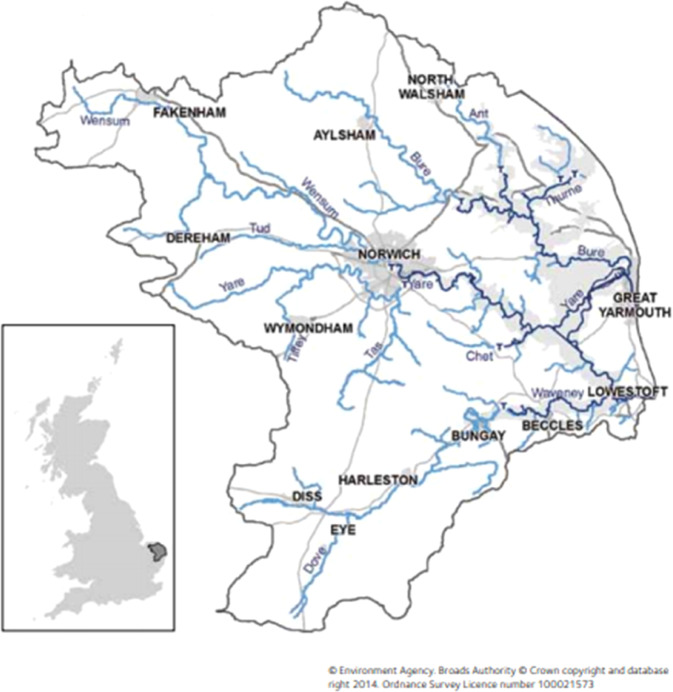
The Broadland Rivers catchment. Source: Environment Agency 2014

(i) Ecosystem services provision has been evaluated both before and after the implementation of PoMs.
  In the case where identification and quantification of ecosystem services delivery have taken place, the analysis should proceed to the next step, whether an ex ante or ex post assessment of the effectiveness of PoMs is concerned.
(ii) Ecosystem services provision has not been assessed or has been assessed for either before or after the implementation of PoMs.
  When PoMs have been developed without any consideration of ecosystem services, identification of ecosystem services can be realised through consultation with stakeholders and/or experts or the deployment of models that are able to assess them. In the case of an ex ante assessment of the effectiveness of PoMs, the expected delivery of ecosystem services can be characterised based on alternative land-use scenarios (Brauman et al. 2011; Egoh et al. 2012; Maes et al. 2013). Other types of analysis such as, ecosystem structure and habitat data (Raffaelli 2006) or functional traits of plants (Lavorel and Grigulis 2012) have been used in the past and could be operationalised for ex post assessments. In addition, catchment stakeholders could engage in this process through participatory workshops (García-Nieto et al. 2015), where they express their knowledge and opinion on the effects of specific PoMs on ecosystem services (Fig. 3).

### Step 3: Assigning Economic Value to Improvements in the Delivery of Ecosystem Services that have Resulted from the Implementation of the PoMs

The changes in the provision of ecosystem services established in the previous step, need now to be evaluated in monetary terms (Saarikoski et al. 2016). Several techniques exist for valuing use (related to actual use of a service) and non-use value (related to passive use of a services) of ecosystems. A strand of the literature has focused on revealed preference methods, such as hedonic pricing (Day 2001), travel cost (Loomis 2003) and averting behaviour; whereas another strand has been concerned with applying stated preference methods, such as the contingent valuation method (Pinto et al. 2016) and choice modelling (Andreopoulos et al. 2015). The choice of the most suitable technique depends on the ecosystem services to be valued (see for example, Reynaud and Lanzanova 2015). In addition, benefit transfer methods, could be cost-effective alternatives. These methods consist of procedures of transferring estimated economic benefit values from a study to a policy site (Plummer 2009). Given that a great number of valuation studies have been performed, benefit transfers started becoming a standard approach in the 1990s (Boutwell and Westra 2013). Although such methods exhibit several shortcomings (Boutwell and Westra 2013), one of the main advantage is the low cost of applying them, since a low volume of site-specific data is not necessary to be gathered.

### Step 4: Comparison of the Results of the Expected or Delivered Value of Ecosystem Services to the Costs of PoMs (Efficiency)

The assessment of the effectiveness of PoMs can now be undertaken through the comparison of the economic value of ecosystem services resulted from the implementation of specific PoMs (Step 3) with the costs of the implemented measures (Step 1). As costs and benefits may be distributed over a number of years, their values need to be turned into current values using a discount rate. This process can evaluate the effectiveness of implemented measures not just in terms of improving water status classification through eliminating significant pressures, but also delivering welfare benefits, when the economic value of eliminating pressures thus improving water status outweighs the costs of the implemented measures.

## Materials and Methods

The Broadlands River catchment, in the UK, was selected as a case study for the application of the ecosystem services framework for assessing the effectiveness of PoMs, to demonstrate how it could be operationalised and utilised by water managers at different river basins. This was based on the availability of information dwelling from the background documents used for the development of the RBMPs.

It should be noted that the following application is only an example that showcases how the methodology could be used to evaluate the effectiveness of selected PoMs. A condition for its application is that the PoMs have been appropriately designed to target catchment pressures to levels that are compatible with the achievement of the ecological objectives introduced by the WFD, or in other words they have been designed properly to deliver water status improvements by minimizing pressures and not just targeting improvements in elements classification as shown to be often the case (Giakoumis and Voulvoulis 2019).

### The Broadlands River Catchment, UK

Broadland Rivers is a part of the Anglian River Basin District studies by the GLOBAQUA project. The Broadland Rivers catchment covers an area of 3200 km^2^ and it is mostly rural. The catchment includes 94 river water bodies with the four main (sub-catchments) being the Bure, Wensum, Yare and Waveney and 19 lake water bodies (Environment Agency 2014).

In 2014, the Broadlands River Catchment Partnership developed a strategic plan for managing key issues in the catchment such as water quality, water quantity, wildlife habitat and recreation. The catchment plan included seven goals and 19 actions related to management of land, water, wastewater, flood risk and sustainable drainage, river and floodplain, recreation and investments to increase funding of the projects (Broadland Catchment Partnership 2014). To achieve these goals and also meet the objectives of the WFD, 84 measures (51 basic and 33 supplementary) were selected during the 1st implementation cycle (Environment Agency 2010, 2015). However, in the end of 2014, the number of water bodies below good status was increased to 108 out of 111, compared to 102 in 2009, indicating that the adopted PoMs were not effective. Giakoumis and Voulvoulis (2019) claim that the reason for this was that managing authorities focused on managing quality elements rather than catchment pressures, treating the symptoms but not the causes.

The completion of the 1st implementation cycle plans was followed by the adoption of measures included in the 2nd management cycle plans. The new and updated measures concerned improving modified physical habitats, managing pollution from wastewater, from urban sources and transport and from rural areas. Overall, 71 policy interventions were considered (Supplementary Tables 7–9).

### Collection of Data

Various sources were used for collecting data (e.g. types and costs of measures, types and importance of ecosystem services, value of ecosystem services, etc.) for in our application of the assessment framework for PoMs in the Broadland Rivers catchment. Information on costs was taken directly from the economic appraisals for the second RBMP of the Anglian Region provided by the Environment Agency^2^ upon request. Data on stakeholders’ perceptions on the connection of local pressures to ecosystem services were gathered during a participative ecosystem service valuation workshop through dynamic group and individual activities (GLOBAQUA 2018). Participants of a Broadland Rivers Catchment Steering Group workshop formed groups and identified 37 ecosystem services as relevant in their catchment. Each stakeholder group was introduced to the concept of ecosystem services and was asked to place each service in the following groups: provisioning, regulating, cultural and supporting services. Information on the possible change of ecosystem services due to the implementation of PoMs was obtained from the catchment summary report (Environment Agency 2014).

### Values Obtained for the Implementation of the Benefit Transfer Approach

After a thorough and detailed review of the economic valuation literature, several studies were selected based on their relevance to the ecosystem services identified for the Broadland Rivers catchment. The Environmental Valuation Reference Inventory^3^ (www.evri.ca) focusing on studies in Europe whenever possible was used, focusing on cases with similar ecosystems and areas with similar socio-economic characteristics, as well as areas that describe similar changes in the status of water resources. While a detailed list of the literature considered for the estimation of the socio-economic value of ecosystem services can be found in Supplementary Table 10, in summary, developing countries with heavily polluted ecosystems were excluded, with most of the studies selected sharing the same policy framework with the Broadland Rivers catchment. The values were estimated using a unit value method and were adjusted for income^4^ and time differences, using data on GDP per capital, purchasing power parity and consumer price index from the World Bank database (https://data.worldbank.org/).

## Results

The application of the framework in the Broadland Rivers catchment, allowed for the assessment of the effectiveness of PoMs selected by the managing authorities to be implemented from 2015 onwards to improve water status by 2021. Results are presented in the following sections that correspond to the steps of the framework for the four sub-catchments of the study area.

### Cost of PoMs Implemented in the Broadland Catchment Area

For the estimation of the PoMs costs, investment costs, administrative and operational costs, resource costs were obtained through the use of hydro-economic modelling (Brouwer et al. 2009), and environmental costs were estimated with the use of market and non-market valuation techniques. For each of the four sub-catchments of the Broadland Rivers catchment, a description of measures and the associated investment and operational costs are presented in Table 1 (more detailed information on the measures can be found in Supplementary Tables 7–9).

**Table 1:** Capital and operational costs of the PoMs in each sub-catchment

Operational catchment	Measures related to	Total length of river (km)	Capital costs	Operational costs (per year)
Yare	Catchment sensitive farming (arable and farming, nutrients); nutrient reduction- phosphate stripping; surface run-off and drainage; enabling fish passage; increasing channel morphological diversity; upgrading existing private sewage systems; channel maintenance strategies; removing obsolete structures and improving sustainable drainage.	158.14	£6282,006.64	£144,555.10
Bure	Catchment sensitive farming (pesticide management); improving in-field grass buffer strips on tillage land and improving riparian buffer strips; enabling fish passage; increasing channel morphological diversity; planting trees; controlling and eradicating of selected high-risk species; supporting established local fora by providing advice and guidance; sharing best practice; increasing awareness of the ‘preventative approach’; improving rural sustainable drainage system within fields, tracks and rural road system; upgrading existing private sewage systems; channel maintenance strategies and removing obsolete structures.	156.03	£6115,410.88	£104,075.68
Waveney	Catchment sensitive farming (arable, farming, livestock, pesticide management, nutrients); nutrient reduction-phosphate stripping; improving in-field grass buffer strips on tillage land and riparian buffer strips; improving rural sustainable drainage system within fields, tracks and rural road system; enabling fish passage; increasing channel morphological diversity and planting trees.	209.26	£9396,387.59	£306,032.07
Wensum	Catchment sensitive farming (arable, farming, livestock, pesticide management, nutrients); nutrient reduction-phosphate stripping; improving in-field grass buffer strips on tillage land and riparian buffer strips; improving rural sustainable drainage system in highway, road, site and housing estate drainage, as well as within fields, tracks and rural road system; enable fish passage; increasing channel morphological diversity; supporting established local fora; share best practice; increase awareness of the ‘preventative approach’ channel maintenance strategies; remove and/or modify obsolete structures; eradication and control of invasive non-native species at selected sites of special scientific interest (SSSI) and Natura 2000 sites and the Wensum restoration strategy.	170.53	£10,796,810.80	£205,887.29

The cost estimates differ across sub-catchment based on the length they are implemented on and/or their type. For example, the costs of measures in Yare and Bure do not vary significantly given that the length that is covered and the types of measures are similar. On the other hand, though measures in Wensum concern similar length of the catchment, a greater number of measures increases the capital and operational costs.

### Changes in the Provision of Ecosystem Services in the Catchment Area as a Result of the PoMs

The connection between selected PoMs and ecosystem services was made through the responses of stakeholders^5^ during a workshop that took place under the GLOBAQUA project^6^. Stakeholders identified the pressures and ecosystem services that are relevant to the Broadland Rivers area. In a recent paper, Giakoumis and Voulvoulis (2018a) analysed the responses and provided the connection between the identified pressures and ecosystem services. Based on their findings, we were able to associate the selected PoMs with the identified ecoystem services through the types of pressures they had been designed to address. Overall, stakeholders neglected supporting services, arguing that such type of services do not provide direct benefits to humans (Haines-Young and Potschin 2012). Therefore, in our analysis, we associated PoMs with these three types of services (Table 2). In addition, since three groups of stakeholders participated in the workshop, we considered the links between pressures and ecosystem services that were recognized by at least two out of the three groups.

**Table 2:** Association between PoMs addressing identified pressures and ecosystem services

Sub-catchment	Measures addresses the following types of pressures	Ecosystem services
Yare	Diffuse and point source pollution	Provisioning (e.g. drinking water)
	Modified habitat	Regulating (e.g. protection from flooding, water purification)
		Recreational (e.g. recreation)
Bure	Diffuse source pollution	Provisioning (e.g. water for irrigation)
	Modified habitat	Regulating (e.g. water purification)
		Recreational (e.g. sense of place)
Wensum	Diffuse and point source pollution	Provisioning (e.g. water for domestic use)
	Modified habitat	Regulating (e.g. soil erosion)
	Other anthropogenic	Recreational (e.g. aesthetic value)
Waveney	Diffuse and point source pollution	Provisioning
	Modified habitat	Regulating (e.g. air quality)
	Other anthropogenic	Recreational (e.g. cultural heritage)

To evaluate changes in ecosystem services from the implementation of the selected PoMs, data from a catchment summary report, published by the Environment Agency (Environment Agency 2014) were used. For each of the main sub-catchments, Table 3 presents the direction (positive/negative) as well as the magnitude of projected impacts of ecosystem services by 2021 as a result of the implementation of PoMs.

**Table 3:** Impact of ecosystem services in the Broadland Rivers catchment accruing from measures implementation

Type of ecosystem services	Sub-catchment			
	Bure	Waveney	Wensum	Yare
Freshwater	+	++	+	+
Food	+			−
Climate regulation	+	++	+	+
Erosion regulation	+	++		+
Water regulation		+	+	+
Water purification and waste treatment		++	+	+
Nutrient cycling		++	+	+
Provision of habitat	+	+	++	+
Aesthetic value		+		
Recreation and tourism	+	++	+	+
Existence values	+			
Cultural heritage		+	−	−

Pluses and minuses express the magnitude of the effect on each ecosystem service (Environment Agency 2014)

The expected changes differ in magnitude across sub-catchment areas, but most of them are positive. In Waveney, the provision of ecosystem services is expected to be increased the highest, whereas in the cases of Wensum and Yare, some services will be negatively impacted. Overall, the information presented above is in line with recent finding concerning the provision of ecosystem services at different water statuses (Grizzetti et al. 2019).

### Value of Expected Changes in the Delivery of Ecosystem Services

For estimating the expected value of changes in ecosystem services in the Broadland Rivers catchment, a benefit transfer approach was used to estimate their value. Using this method, the following results were obtained (Table 4).

**Table 4:** Type of services and range of values obtained from the benefit transfer application

Type of service	Range of values (£/person/year)
Provisioning	1.96–15.54
Regulating	1.23–72.88
Cultural	0.48–23.20

The variation in the values can be attributed to the fact that the selected studies included both finite and infinite time horizons. To reduce uncertainty^7^ in monetary estimates, a number of studies per ecosystem service was used, and the time horizon of each selected study was considered. The final estimates included in the analysis were average values of all estimates per selected study. By considering the number of individuals affected by the policy interventions yielded the following estimates per operational catchment.

The aggregated value of ecosystem services was calculated by considering the population in each sub-catchment. This approach does not account for preference heterogeneity that among others may be related to the distance of the stakeholders from the resource (Bateman et al. 2006), therefore the obtained values may be overestimated due to transfer errors (Johnston and Rosenberger 2009; Ready and Navrud 2006). For this reason, we accounted for a transfer error of 70%, which is within the transfer error estimates recognized by relevant studies (Stellin and Candido 2006) (Table 5).

**Table 5:** Value of ecosystem services in the four operational catchments

Category	Bure	Waveney	Wensum	Yare	Discounted total (40-year period, 3.5% discount rate)
Provisioning (e.g. consumption of water for domestic and agricultural use)	£50,231,898	£122,391,854	£50,725,269	£54,212,506	£277,561,526
Regulating (e.g. flood and erosion control)	£104,673,371	£353,405,720	£162,345,138	£166,842,335	£787,266,564
Cultural (e.g. recreation, landscape beauty, sense of place)	£38,089,579	£140,149,944	£59,075,751	£60,712,235	£298,027,509

### Comparison of Expected Costs and Benefits due to Implementation of PoMs

The values obtained in Step 4 were aggregated given the number of inhabitants estimated using the area of each river catchment and the population density obtained from Office for National Statistics published data (Office for National Statistics 2011). The following formula was used to estimate the net present value of cost:

$$\mathop {\sum}\limits_{t,i}^{T,N} {\frac{{w_i\widehat B_{i,t} - \widehat C_t}}{{\left( {1 + r} \right)^t}}}$$

where $\widehat B$ and $\widehat C$ are the estimated benefits and costs*, w* is the probability of obtaining the estimated benefits and *r* the discount rate. The time horizon used for the estimation of benefits is 40 years and the success rate of PoMs was 70%, similar to that included in the economic appraisal of measures of the Environment Agency (Table 6).

**Table 6:** Net present value of benefits and costs (£ in 2015) incorporating the value of ecosystem services in the Broadland Rivers basin sub-catchments

Sub-catchment	Present value of costs	Present value of benefits	Net present value	Benefit–cost ratio
Discount rate: 6%				
Yare	£8457,026	£139,718,116	£131,261,091	16.5
Bure	£7681,365	£95,652,582	£87,971,217	12.5
Waveney	£14,001,037	£163,461,551	£149,460,514	11.7
Wensum	£13,894,652	£134,953,270	£121,058,618	9.7
Catchment total	£44,034,079	£533,785,519	£489,751,440	12.1
Discount rate: 4.5%				
Yare	£8942,050	£170,279,403	£161,337,353	19.0
Bure	£8030,568	£116,611,701	£108,581,133	14.5
Waveney	£15,027,863	£199,222,156	£184,194,293	13.3
Wensum	£14,585,463	£164,467,755	£149,882,292	11.3
Catchment total	£46,585,944	£650,581,014	£603,995,071	14.0
Discount rate: 3.5%				
Yare	£9368,991	£197,236,953	£187,867,962	21.1
Bure	£8337,955	£135,096,394	£126,758,439	16.2
Waveney	£15,931,725	£230,765,460	£214,833,735	14.5
Wensum	£15,193,549	£190,502,311	£175,308,762	12.5
Catchment total	£48,832,219	£753,601,117	£704,768,898	15.4

As the temporal distribution of benefits and costs is not known, a sensitivity analysis was undertaken (for more information, see for example, Pearce et al. 2006; Moore et al. 2004) to investigate how these would respond to different discount rates (6, 4.5 and 3.5%). Under every scenario, the benefits of implementing the selected water measures were found to be higher than the relevant costs.

Benefits and costs were not found to be distributed uniformly across the four sub-catchments. Regulating services seem to obtain the highest value in every sub-catchment. This is in line with earlier results of similar estimation exercises (e.g. Koundouri et al. 2015; Costanza et al. 2006). Cultural services are less valued, which contradicts the results of other studies (Ghermandi et al. 2010); however, this should be attributed to the magnitude of change in each area, as well as negative impacts on sights of significant cultural and aesthetic importance due to the implementations of PoMs. Another reason associated with this might be that though river landscape incorporate high aesthetic and cultural value (Thiele et al. 2019), it is difficult to quantify such non-material values and associate them with alterations in ecosystems (Verbrugge et al. 2019). Second, the total value of benefits is influenced by the population in each area, as well as the expected magnitude of change of the provision of ecosystem services. The ecosystem services are expected to be affected the most in Waveney, where according to our estimate, the majority of the population is located. As a consequence, the highest values of benefits are associated with Waveney under each scenario.

Besides that, the benefit–cost ratios seem significantly higher than those included in the catchment summary report, which present benefit–cost ratios ranging from 1.24 to 4.9. This can be attributed to two factors. The first is that though supporting services were excluded from our analysis, the specific subcategories of ecosystem services associated with significant pressures in the area reported by the stakeholders are broader than that included in the catchment summary report that mostly was the result of expert opinions. Therefore, if the values presented in this study are not subject to transfer error higher than 70%, stakeholders’ participation may result in capturing a wider range of benefits. This means that had the PoMs designed to address pressures and not improved the elements classification, the net benefit resulted from water status enhancement would be higher than that reported in the catchment report. Second, the cost estimates also differ. As cost data were taken from official sources, it is not clear why these values are different from those reported in the catchment report, nor what kind of cost elements (economic, environmental, resource) are included in each case.

Finally, the costs are higher in sub-catchments with higher number of water bodies, where more interventions take place. In addition, the costs of PoMs for Bure, Wensum and Waveney that include a mix of technical and non-technical measures (e.g. sharing best practices) are relatively lower than the PoMs for Yare as they consist mainly of technical interventions. In terms of financing, this might mean that when capital is a significant constraint, and the ecological status of water is not heavily impacted, non-technical measures that focus on the changing of stances in relation to water resources might be an alternative cost-effective route.

## Discussion

Achieving the water status classification objectives of the WFD requires in depth understanding of the interactions between the natural and social systems (Voulvoulis et al. 2017). Managing authorities require knowledge of the sub-systems embodied in each catchment and developing management tools that are able to influence how these sub-systems interact with one another. In support of this, the WFD adopted the Drivers–Pressures–State–Impacts–Responses framework (Commission 2003). As a result, when implementing the directive, Member States need to assess the gap between the current status and optimal environmental conditions defined as a status, where pressures are absent or unable to affect water quality (Voulvoulis et al. 2017). Consequently, the main purpose of PoMs is to alleviate identified pressures and their effects on waters. In addition, effective PoMs, need to be able to achieve an equilibrium among various often conflicting objectives related to these sub-systems, ultimately reducing the gap to good water status.

Deciding on the most suitable measures should be based on information on their ability to tackle pressures as well as on their costs. The WFD necessitates the use of economic principles and techniques to assess their effectiveness. In many instances, CEA has been used for this procedure; however, the literature recognizes several issues (Martin-Ortega 2012; Messner 2006). First, CEA might neglect social aspects of water status improvements, which might impact the actual implementation of policies. Second, the costs of measures may exhibit nonlinearities and may be space-, time- and scale-specific, which makes comparisons of CEA results problematic. In addition, measures may have indirect side-effects on ‘separate spheres’ (Brock 2003), which may be beyond the scope of the environmental problem they aim to tackle. Another method that has also been suggested by the European Commission and overcomes some of the significant flaws of CEA is CBA, but its application requires caution as it is conditional to the appropriate design on PoMs. CBA assigns monetary values to direct and indirect costs and benefits of policy intervention and can be used for assessing the economic efficiency of environmental policies both ex ante and ex post. In the core of CBA is the assessment of whether an environmental policy results in achieving the desired objective, while improving social well-being by generating use and non-use benefits (Hanley et al. 2006; Hanley and Black 2006). As CEA, CBA has also received criticism. According to Sunstein (2005), WTP is not always an appropriate measure, as through it, citizens may express their judgements instead of their preferences. Furthermore, given biases embodied in the valuation methods used to elicit economic value, philosophical discussions about CBA focus on the difficulty of assigning economic value to what is conceived as invaluable (Hansson 2007), but the use of ecosystem services alleviates this challenge to an extent.

The 5th Implementation Report states that 11 out of the total number of EU Member States developed some kind of CBA (European Commission 2019) and though steps were made in performing economic analysis, significant gaps still exist in translating the results of economic analysis in measures, thus follow more integrated water management approaches. Member States have been focusing more on complying with the requirements of the Directive, than harvesting the true potential of the WFD (Behagel 2012; Giakoumis and Voulvoulis 2018b; Petersen et al. 2009). This fact has been expressed through the insignificant increase in the number of water bodies whose status improved after the implementation of measures. In line with this, the current study presents a case where though the economic analysis of selected measures was sophisticated and took into account environmental aspects, the results in terms of enhancing waters have been discouraging, demonstrating the importance of developing appropriate PoMs. Unless the environmental problems are framed properly in terms of pressures and impacts, there is little hope in evaluating the effectiveness of measures when their application does not deliver overall status improvements.

Recognizing this, as well as the results of a previous study (Giakoumis and Voulvoulis 2018a), the methodology presented in this paper describes a way of assessing PoMs by connecting pressures to measures and ecosystem services. In the heart of the methodology lies the idea that improvement in the water status results from mitigating pressures on water. Consequently, following the spirit of Radev et al. (2020), our methodology requires the selected combination of measures to enhance water status. Making use of the principles of CBA for assessing the effectiveness of measures, we incorporate ecosystem services into the analysis to achieve a straightforward connection between the costs and benefits (social and environmental) of policy measures and the impacts on the well-being of relevant stakeholders. The use of ecosystem services in the assessment of policies facilitates a connection between the environmental and social systems (Maes et al. 2018) and comprehensive communication of the benefits of effective implementation of the WFD, thus has the potential to promote commitment to policy decision (Howarth 2009). In addition, it provides a systemic view of the nature–society relationship (Voulvoulis 2012) and decreases the risk of adopting traditional standardised practices that are not related to the catchment (Sabatier et al. 2005). Last, it enables managers to address multiple goals (Everard 2014), which could have added benefits for the European Union, where environmental management practices are defined by extensive legislation for the different aspects of environmental systems (Beunen et al. 2009; Bouwma et al. 2018; Jordan and Lenschow 2010; Schleyer et al. 2015). Here we demonstrated how the methodology for assessing the effectiveness of PoMs can be applied prior to the implementation of measures. Its application is information-intensive, as several types of data are needed for fulfilling its steps. Costs of PoMs are an essential part of the analysis, therefore such estimates should either be collected through WFD documents or be estimated. In addition to that, in order to harvest the benefits of this methodology, data on ecosystem services provision are essential. However, as the adoption of this concept is a growing trend in several countries (e.g. the UK National Ecosystem Assessment (Watson et al. 2011), Spanish National Ecosystem Assessment (Fundación Biodiversidad 2014), Portuguese Millennium Ecosystem Assessment (Pereira et al. 2009)), as well as in scientific projects funded by the EU (e.g. GLOBAQUA, MARS, OpenNESS), such information might already be available in several EU countries. Concerning the estimation of the value of ecosystem services, in our empirical example we applied a Benefit Transfer method. Due to possible transfer errors (Boutwell and Westra 2013; Johnston et al. 2018; Kaul et al. 2013), primary studies (e.g. hedonic pricing and choice experiments) should be used in cases where capital and time constraints are less strict.

The unexpected high benefit–cost ratios obtained in our case study reveal the importance of developing the measures appropriately before applying the economic analysis. In other words, there is a clear risk in evaluating measures that do not mitigate pressures but return high benefit–cost ratios in the assessment. It should be highlighted therefore, that the methodology does not assess whether or not adopted measures are able to ensure water status improvements but compares measures that have been designed to address pressures and deliver status improvements in terms of benefit–cost ratios. Furthermore, the application in the Broadland Rivers catchment demonstrated how the methodology can also accommodate stakeholder’s participation in the assessment of environmental policies and through them, reveal the impacts of improvements of the status of natural resources.

## Conclusion

The proposed methodology provides a holistic way for water managers to assess the effectiveness of PoMs, while making sure that local stakeholder opinions are considered, and measures do not yield disproportionally high costs in relation to benefits accruing from improving water status. The proposed assessment framework could benefit water management practitioners to frame environmental problems more accurately and assess the effects of their practices in a more systemic manner. It can be used either in the initial process of selecting cost-effective measures to provide insight of their socio-economic impact, or after the implementation of measures to validate whether they have been economically beneficial or not. In addition, it could be used after the conclusion of a management cycle, to assess whether implemented actions have been effective or not.

Finally, our study presents a possible way to integrate different kinds of knowledge (e.g. biology, sociology, economics, ecology, etc.) into a common framework. Economists or ecologists would most likely fail to understand the mechanics of the suggested methodology as well as obtain sound results if they only focused on the assumptions, methods and research practices of their own discipline. Therefore, the implementation of the suggested methodology requires both collaboration among experts of various fields as well as understanding of how different disciplines distinguish themselves.

## Supplementary information

Supplementary Materials

## Data Availability

All data sources used are appropriately cited. Original model files are available from the authors upon request.

## Footnotes

1 Methodological and practical issues of the two method are further presented in the last section of this article.

2 Public sector information licensed under the Open Government Licence v3.0.

3 The Environmental Valuation Reference Inventory is a databank that contains empirical studies on the economic value of environmental assets and human health.

4 Following the study of Koundouri et al. (2014), we used an income elasticity of demand for the environmental good in question of 0.5.

5 Among the participants were representatives of the Broadland Catchment Partnership, the Broads Authority, the Anglia Ruskin University, the Anglian Water Services Limited, the Country Land & Business Association Limited, the Department for Environment, Food & Rural Affairs, the Environment Agency, the Northumbrian Water Ltd, the National Farmers Union, the National Trust, the Natural England body, the Norfolk County Council, the Norfolk Wildlife Trust, the River Waveney Trust, the Rivers Trust, the Suffolk Wildlife Trust, the University of East Anglia, the Society for the Protection of Birds and the Water Management Alliance.

6 Methodology and results of ecosystem services co-definition and participative valuation workshop are described on GLOBAQUA (2017).

7 The term relates to the uncertainty embodied in the economic estimates of relevant economic studies.
