# Supplementary material for: Incorporating Ecosystem Services in the Assessment of Water Framework Directive Programmes of Measures
Source: Environ Manage. 2021 May 12;68(1):38–52. doi: 10.1007/s00267-021-01478-7 (PMC8172509; doi:10.1007/s00267-021-01478-7)
Supplement: Supplementary file 1 — Supplementary Materials [file 267_2021_1478_MOESM1_ESM.docx]

# Incorporating ecosystem services in the assessment of Water Framework Directive programmes of measures

I.Souliotis^a^, N. Voulvoulis*^a^

^a^Centre for Environmental Policy, Imperial College London, London SW7 2AZ, United Kingdom

*Corresponding author. Tel.: +44 020 7594 7459; Fax: +44 020 7594 9334.

Email: n.voulvoulis@imperial.ac.uk

**Supplementary Material**

Supplementary material: Table 7, Table 8, Table 9

Table 7, Table 8 and Table 9 contain a list of measures that have been selected to improve water status either by 2021 or beyond 2027 in the Broadland Rivers catchment. The information was provided by the Environment Agency (Environment Agency 2015)

Table 7 List of measures which will happen by 2021 and achieve environmental outcomes, but there is not enough confidence (in location or scale of improvement) to predict specific outcomes. Such measures are described in section 3.3 Part 1 of the Anglian river basin management plan.

| **No. of measure** | **Operational Catchment** | **Measure category** | **Description of measure** | **Estimated start date** | **Sector of lead organisation** | **Key Type of Measure** |
| --- | --- | --- | --- | --- | --- | --- |
| 1 | Wensum | To improve modified habitat | Habitat improvement - Wensum tributaries | 1/7/2015 | Environment, Farming, Rural | KTM6 - Improving hydromorphological conditions of water bodies other than longitudinal continuity |
| 2 | Waveney | To improve modified habitat | Habitat restoration - Waveney habitat project | 1/7/2015 | Environment, Farming, Rural | KTM6 - Improving hydromorphological conditions of water bodies other than longitudinal continuity |
| 3 | Bure | To control or manage diffuse source inputs | Reduce diffuse pollution pathways - "Broadland Slow the Flow" project | 1/7/2015 | Environment, Farming, Rural | KTM2 - Reduce nutrient pollution from agriculture |
| 4 | Waveney | To control or manage point source inputs | Additional treatment to reduce concentrations of nutrients 5from Pulham St Mary STW | 31/3/2020 | Waste water treatment | KTM1 - Construction or upgrades of wastewater treatment plants |
| 5 | Waveney | To control or manage point source inputs | Additional treatment to reduce concentrations of phosphate from Hoxne sewage treatment works. | 31/3/2020 | Waste water treatment | KTM1 - Construction or upgrades of wastewater treatment plants |
| 6 | Waveney | To control or manage abstraction | Change in abs lic condtn(s) to address potential serious damage at full license, Dickleburgh Stream | 26/6/2015 | Environment, Farming, Rural | KTM6 - Improving hydromorphological conditions of water bodies other than longitudinal continuity |
| 7 | Wensum | To control or manage abstraction | Change in location of abstraction | 1/7/2015 | Environment, Farming, Rural | KTM6 - Improving hydromorphological conditions of water bodies other than longitudinal continuity |

Table 8 A summary of the additional measures needed to achieve objectives beyond 2021. The measures listed here were used to produce the summary programmes of measures in table 22 in section 3.5 of Part 1 of the river basin management plan.

| **No. of measure** | **Operational catchment** | **Bundle** | **Measure category 1** | **Measure category 2** | **Measure category 3** |
| --- | --- | --- | --- | --- | --- |
| 8 | Bure | G1 (to good status bundle, cost beneficial) | To improve modified habitat | Removal or easement of barriers to fish migration | Enable fish passage (e.g. fish pass) |
| 9 | Bure | G1 (to good status bundle, cost beneficial) | To improve modified habitat | Improvement to condition of channel/bed and/or banks/shoreline | Increase in-channel morphological diversity |
| 10 | Bure | G1 (to good status bundle, cost beneficial) | To control or manage diffuse source inputs | Reduce diffuse pollution pathways (i.e. control entry to water environment) | Surface run-off & drainage management |
| 11 | Bure | G1 (to good status bundle, cost beneficial) | To control or manage diffuse source inputs | Reduce diffuse pollution pathways (i.e. control entry to water environment) | Surface run-off & drainage management |
| 12 | Bure | G1 (to good status bundle, cost beneficial) | To control or manage diffuse source inputs | Reduce diffuse pollution at source | Field & Crop - Arable soils |
| 13 | Bure | G1 (to good status bundle, cost beneficial) | To control or manage diffuse source inputs | Reduce diffuse pollution at source | Field & Crop - Livestock |
| 14 | Bure | G1 (to good status bundle, cost beneficial) | To control or manage diffuse source inputs | Reduce diffuse pollution pathways (i.e. control entry to water environment) | Surface run-off & drainage management |
| 15 | Bure | G1 (to good status bundle, cost beneficial) | To control or manage non native invasive/alien species | Building awareness and understanding (to slow the spread) | Implement Individual Species Action Plans for priority species. |
| 16 | Bure | G1 (to good status bundle, cost beneficial) | To control or manage non native invasive/alien species | Mitigation, control and eradication (to reduce extent) | Share best practice on partnership working |
| 17 | Waveney | A (alternative objective bundle, cost beneficial) | To control or manage point source inputs | Mitigate/Remediate point source impacts on receptor | Install nutrient reduction |
| 18 | Waveney | A (alternative objective bundle, cost beneficial) | To improve modified habitat | Removal or easement of barriers to fish migration | Enable fish passage (e.g. fish pass) |
| 19 | Waveney | A (alternative objective bundle, cost beneficial) | To improve modified habitat | Improvement to condition of channel/bed and/or banks/shoreline | Increase in-channel morphological diversity |
| 20 | Waveney | A (alternative objective bundle, cost beneficial) | To control or manage diffuse source inputs | Reduce diffuse pollution pathways (i.e. control entry to water environment) | Surface run-off & drainage management |
| 21 | Waveney | A (alternative objective bundle, cost beneficial) | To control or manage diffuse source inputs | Reduce diffuse pollution pathways (i.e. control entry to water environment) | Surface run-off & drainage management |
| 22 | Waveney | A (alternative objective bundle, cost beneficial) | To control or manage diffuse source inputs | Reduce diffuse pollution at source | Field & Crop - Nutrients |
| 23 | Waveney | A (alternative objective bundle, cost beneficial) | To control or manage diffuse source inputs | Reduce diffuse pollution pathways (i.e. control entry to water environment) | Surface run-off & drainage management |
| 24 | Waveney | A (alternative objective bundle, cost beneficial) | To improve modified habitat | Vegetation management | Plant new vegetation |
| 25 | Waveney | A (alternative objective bundle, cost beneficial) | To control or manage diffuse source inputs | Reduce diffuse pollution at source | Field & Crop - Arable soils |
| 26 | Waveney | A (alternative objective bundle, cost beneficial) | To control or manage diffuse source inputs | Reduce diffuse pollution at source | Field & Crop - Livestock |
| 27 | Waveney | A (alternative objective bundle, cost beneficial) | To control or manage diffuse source inputs | Reduce diffuse pollution at source | Field & Crop - Pesticide management |
| 28 | Waveney | A (alternative objective bundle, cost beneficial) | To control or manage non native invasive/alien species | Early detection, monitoring and rapid response (to reduce the risk of establishment) | Control and eradication of selected high risk species |
| 29 | Waveney | A (alternative objective bundle, cost beneficial) | To control or manage non native invasive/alien species | Mitigation, control and eradication (to reduce extent) | Share best practice on partnership working |
| 30 | Waveney | A (alternative objective bundle, cost beneficial) | To control or manage non native invasive/alien species | Building awareness and understanding (to slow the spread) | Implement Individual Species Action Plans for priority species. |
| 31 | Waveney | A (alternative objective bundle, cost beneficial) | To control or manage non native invasive/alien species | Mitigation, control and eradication (to reduce extent) | Support established local fora by providing advice and guidance |
| 32 | Wensum | G1 (to good status bundle, cost beneficial) | To improve modified habitat | Improvement to condition of channel/bed and/or banks/shoreline | Increase in-channel morphological diversity |
| 33 | Wensum | G1 (to good status bundle, cost beneficial) | To improve modified habitat | Removal or easement of barriers to fish migration | Enable fish passage (e.g. fish pass) |
| 34 | Wensum | G1 (to good status bundle, cost beneficial) | To control or manage diffuse source inputs | Reduce diffuse pollution pathways (i.e. control entry to water environment) | Surface run-off & drainage management |
| 35 | Wensum | G1 (to good status bundle, cost beneficial) | To control or manage diffuse source inputs | Reduce diffuse pollution pathways (i.e. control entry to water environment) | Surface run-off & drainage management |
| 36 | Wensum | G1 (to good status bundle, cost beneficial) | To control or manage diffuse source inputs | Reduce diffuse pollution at source | Field & Crop - Nutrients |
| 37 | Wensum | G1 (to good status bundle, cost beneficial) | To control or manage diffuse source inputs | Reduce diffuse pollution at source | Field & Crop - Arable soils |
| 38 | Wensum | G1 (to good status bundle, cost beneficial) | To control or manage diffuse source inputs | Reduce diffuse pollution pathways (i.e. control entry to water environment) | Surface run-off & drainage management |
| 39 | Wensum | G1 (to good status bundle, cost beneficial) | To control or manage diffuse source inputs | Reduce diffuse pollution pathways (i.e. control entry to water environment) | Surface run-off & drainage management |
| 40 | Wensum | G1 (to good status bundle, cost beneficial) | To control or manage diffuse source inputs | Reduce diffuse pollution at source | Field & Crop - Pesticide management |
| 41 | Wensum | G1 (to good status bundle, cost beneficial) | To control or manage diffuse source inputs | Reduce diffuse pollution at source | Field & Crop - Livestock |
| 42 | Wensum | G1 (to good status bundle, cost beneficial) | To control or manage non native invasive/alien species | Mitigation, control and eradication (to reduce extent) | Share best practice on partnership working |
| 43 | Wensum | G1 (to good status bundle, cost beneficial) | To control or manage non native invasive/alien species | Building awareness and understanding (to slow the spread) | Implement Individual Species Action Plans for priority species. |
| 44 | Yare | G1 (to good status bundle, cost beneficial) | To improve modified habitat | Removal or easement of barriers to fish migration | Enable fish passage (e.g. fish pass) |
| 45 | Yare | G1 (to good status bundle, cost beneficial) | To improve modified habitat | Improvement to condition of channel/bed and/or banks/shoreline | Increase in-channel morphological diversity |
| 46 | Yare | G1 (to good status bundle, cost beneficial) | To control or manage diffuse source inputs | Reduce diffuse pollution pathways (i.e. control entry to water environment) | Surface run-off & drainage management |
| 47 | Yare | G1 (to good status bundle, cost beneficial) | To control or manage diffuse source inputs | Reduce diffuse pollution pathways (i.e. control entry to water environment) | Surface run-off & drainage management |
| 48 | Yare | G1 (to good status bundle, cost beneficial) | To control or manage diffuse source inputs | Reduce diffuse pollution at source | Field & Crop - Nutrients |
| 49 | Yare | G1 (to good status bundle, cost beneficial) | To control or manage diffuse source inputs | Reduce diffuse pollution at source | Field & Crop - Livestock |
| 50 | Yare | G1 (to good status bundle, cost beneficial) | To control or manage diffuse source inputs | Reduce diffuse pollution at source | Field & Crop - Arable soils |
| 51 | Yare | G1 (to good status bundle, cost beneficial) | To improve modified habitat | Removal or modification of engineering structure | Remove structures |
| 52 | Yare | G1 (to good status bundle, cost beneficial) | To control or manage diffuse source inputs | Reduce diffuse pollution pathways (i.e. control entry to water environment) | Surface run-off & drainage management |
| 53 | Yare | G1 (to good status bundle, cost beneficial) | To control or manage point source inputs | Mitigate/Remediate point source impacts on receptor | Install new private STW |
| 54 | Yare | G1 (to good status bundle, cost beneficial) | To control or manage point source inputs | Mitigate/Remediate point source impacts on receptor | Upgrade existing private STW |

Table 9 Summary of the programmes of measures that will improve the water environment by 2021. The estimated starting date for most of these measures is set to be 2015. These main programmes are described in section 3.3 of Part 1 of the river basin management plan.

| **No of measure** | **Water body ID/ name** | **Measure category 1** | **Description of outcome** | **Key Type of Measure** |
| --- | --- | --- | --- | --- |
| 55 | GB105034055882 | To improve modified habitat | To prevent eels and elvers from being entrained (sucked into) river abstractions and prevented from returning upstream by obstructions, the Eels Regulations require appropriate screening to be fitted to abstractions and obstructions to be removed or by-passed. These measures should not only prevent entrainment of eels, but also other fish species. | KTM6 - Improving hydro morphological conditions of water bodies other than longitudinal continuity |
| 56 | GB105034055882 | To improve modified habitat | To prevent eels and elvers from being entrained (sucked into) river abstractions and prevented from returning upstream by obstructions, the Eels Regulations require appropriate screening to be fitted to abstractions and obstructions to be removed or by-passed. These measures should not only prevent entrainment of eels, but also other fish species. | KTM6 - Improving hydro morphological conditions of water bodies other than longitudinal continuity |
| 57 | GB105034051281 | To improve modified habitat | To prevent eels and elvers from being entrained (sucked into) river abstractions and prevented from returning upstream by obstructions, the Eels Regulations require appropriate screening to be fitted to abstractions and obstructions to be removed or by-passed. These measures should not only prevent entrainment of eels, but also other fish species. | KTM6 - Improving hydro morphological conditions of water bodies other than longitudinal continuity |
| 58 | GB30536989 GB30547009 GB105034055730 GB105034055881 | To control or manage diffuse source inputs | prevent deterioration, or contribute to the achievement of protected area objectives, reduce the impact of diffuse pollution that arises from rural land use | KTM23 - Natural water retention measures |
| 59 |  | To improve modified habitat | River restoration works to improve hydro morphology and diffuse pollution on the urban Clipstone Brook | KTM6 - Improving hydro morphological conditions of water bodies other than longitudinal continuity |
| 60 | Catchment wide | To improve modified habitat | Broadland Catchment Partnership will provide multiple benefits, joining the resources of a range of organisations towards delivery of WFD objectives. This project will allow the delivery of an action plan published in 2014, and is supported financially by a range of partners from public, private and third sectors. The Broadland Catchment Plan identifies seven main goals, including reducing flood risk and promoting sustainable drainage. The Partnership will work with Norfolk County Council using the mapping of surface water flooding risk across the county. It will work with landowners and highways to reduce flood risk. Several actions in the Plan are already underway, in particular those for land management. By continuing to contribute to the Broadland Catchment Partnership we can help ensure these actions and others are completed. Specific focus this year will be given to setting up demonstration projects for rural drainage in high run off areas and exploring potential locations for constructed wetlands to reduce phosphorus downstream of urban areas. | KTM6 - Improving hydro morphological conditions of water bodies other than longitudinal continuity |
| 61 | Catchment wide | To improve modified habitat | This is partnership project with Rochford District Council and the Essex Wildlife Trust will restore a Heavily Modified Water Body to a more natural state and address water quality issues from both urban and rural areas. The section of Nobles Green Ditch where this project will be undertaken encompasses a Country Park downstream of a large sewage works. As well as being impacted by urban influences and road runoff this water course is also impacted by the surrounding land. The project will entail in-channel and riparian habitat improvements as well as removing a barrier currently impassable to fish. This project will lead to an improved ecological status for this water body. | KTM6 - Improving hydro morphological conditions of water bodies other than longitudinal continuity |
| 62 | Catchment wide | To control or manage diffuse source inputs | The catchment of the Yare is largely agricultural, but also passes through Wymondham and southern suburbs of Norwich in its lower reaches. Both towns can cause urban pollution from Combined Sewer Overflows and misconnections. Road drainage in parts of the catchment acts as a conduit for sediment borne pollution, a particular problem on narrow rural roads where verges are undermined and sediment is lost from field gates. Many protected areas are fed by these rivers and receive nutrient enrichment. Parts of the catchment is designated under the Habitats Directive and subject to Diffuse Water Pollution Plans to improve the status of these designated sites. Measures identified in these plans include the reduction of run off from highways.  We will use the output from a project that identified sediment pathways to the rivers from highways sources to identify highway drainage improvements. This will include sediment trapping in suitable locations where landowners are willing. We will also work with Norfolk County Council as they develop their flood risk strategy and surface water management plans to identify mutually agreeable options for attenuating flood water from urban areas and highways sources. | KTM21 - Measures to prevent or control the input of pollution from urban areas, transport and built infrastructure |
| 63 | Area wide | To improve modified habitat | Several of our water bodies are failing for fish, plants and invertebrates. Tree planting, particularly in the headwaters, will provide shading; cooling the water temperature which will benefit fish spawning, invertebrates and reduce macrophyte growth. Working with a number of landowners, Catchment Partnerships and other partners across the catchments of our three counties we will undertake several tree planting projects which will achieve these benefits as well as reducing agricultural run-off from entering the water courses. | KTM6 - Improving hydro morphological conditions of water bodies other than longitudinal continuity |
| 64 |  | To improve modified habitat | This project will implement the mitigation measures improving habitats required under WFD, alongside other locally identified opportunities. | KTM6 - Improving hydro morphological conditions of water bodies other than longitudinal continuity |
| 65 |  | To improve modified habitat | Wetland creation to reduce nutrient input and levels in the River Glaven upstream of recent river restoration works. | KTM6 - Improving hydro morphological conditions of water bodies other than longitudinal continuity |
| 66 |  | To improve modified habitat | Urban river morphology project involving local community, volunteers, Ipswich Borough Council and Suffolk County Council.  A detailed plan has been produced by Environment Agency in collaboration with partners | KTM6 - Improving hydro morphological conditions of water bodies other than longitudinal continuity |
| 67 | GB104028053340, GB104028042490, GB104028042501, GB104028042502, GB104028042510, GB104028042520, GB104028046430, GB104028042640, GB104028053310, GB104028053380, GB104028042400, GB104028046680, GB104028046840, GB104028047030, GB104028053110, GB104028053250, GB104028064290, GB109054044140, GB109054044520, GB109054044660, GB109054049144, GB70410266, GB70410508, GB70410537, GB70910519, GB71210541 | To improve modified habitat | Habitat restoration in headwaters of River Waveney | KTM6 - Improving hydro morphological conditions of water bodies other than longitudinal continuity |
| 68 |  | To improve modified habitat | Improvements to riparian habitat, tree planting, reconnecting flood plain, reconnecting old river channel and bank re-profiling. Working with Chelmsford City Council Parks It is hoped to re-designate the park as a Local Nature Reserve, and provide an on-going maintenance regime to enhance it’s biodiversity value. | KTM6 - Improving hydro morphological conditions of water bodies other than longitudinal continuity |
| 70 |  | To control or manage diffuse source inputs | Sediment laden run-off from land informally used by 4x4 vehicles is causing water quality issues and habitat degradation at two locations in the Chelmer catchment, one of which is affecting a local wildlife site.  Rural Sustainable Urban Drainage systems are to be used to trap sediment and improve habitat, whilst land damaged by the 4x4 activity will be restored and security improved to help prevent un-authorised vehicle access. | KTM17 - Measures to reduce sediment from soil erosion and surface run-off |
| 71 |  | To control or manage diffuse source inputs | The project is led by the Game and Wildlife Conservation Trust and the Freshwater Habitats Trust. The Water Friendly Farming project in the upper Welland takes a rigorous approach to implementation of resource protection measures, with two ‘treatment’ catchments and a comparable control catchment. The project focuses heavily on soil and land use management, and delivers a balance between research and development, and practical implementation of a wide variety of best practice land management techniques and technology trials. The project aims to reduce sediment loss, and associated diffuse pollution to watercourses, which impacts on the ecology, amenity use, and often requires removal of sediment by use of public funds in the lower sections of the catchment. The Water Friendly Farming project will help us further understand how to achieve effective integrated catchment management; providing both environmental and flood risk benefits, with results and methodology that can be applied to other catchments where necessary and appropriate. The project is ongoing with annual investment since at least 2011 from landowners, academic institutes, Anglian Water and private sector agricultural organisations. | KTM17 - Measures to reduce sediment from soil erosion and surface run-off |

**Table 10** Studies used for the estimation of the ecosystem services benefits from improvements in the Broadland Rivers catchment.

| **Study** | **Country** | **Type of services** |
| --- | --- | --- |
| Adamowicz et al. (1995) | UK | Cultural |
| Ahtiainen et al. (2014) | Finland | Regulating |
| Alcon et al. (2013) | Spain | Regulating |
| Barrio and Loureiro (2013) | Spain | Cultural |
| Bateman et al. (2006) | UK | Provisioning |
| Birol et al. (2013) | Poland | Regulating |
| Brouwer and Bateman (2005) | UK | Regulating |
| Castro et al. (2016) | USA | Cultural, regulating, provisioning |
| Doherty et al. (2014) | Ireland | Cultural, regulating |
| Bouscasse et al. (2011) | France | Provisioning |
| Genius et al. (2008) | Greece | Provisioning |
| Genius et al. (2012) | Greece | Provisioning |
| He et al. (2017) | Canada | Regulating |
| Hein (2011) | Netherlands | Provisioning |
| Koundouri et al. (2014) | Greece | Provisioning |
| Markantonis et al. (2013) | Greece | Regulating |
| Polyzou et al. (2011) | Greece | Provisioning |
| Stithou et al. (2012) | Ireland | Cultural, regulating |

**References**

Adamowicz, WL, G Garrod, and KG Willis. 1995. Development *Estimating the Passive-Use Benefits of Britain’s Inland Waterways*. https://scholar.google.gr/scholar?hl=en&as_sdt=0%2C5&q=Estimating+the+passive+use+benefits+of+Britain%27s+inland+waterways.+Centre+for+Rural+Economy%2C+Department+of+Agricultural+Economics+and+Food+Marketing%2C+University+of+Newcastle+upon+Tyne.&btnG= (May 9, 2019).

Ahtiainen, Heini, Janne Artell, and Eija Pouta. 2014. “Using Individual-Specific Status Quo Alternative in Choice Experiments: Heterogeneous Preferences for Water Quality.” In *5th World Congress of Environmental and Resource Economists*,.

Alcon, F et al. 2013. “The Non-Market Value of Reclaimed Wastewater for Use in Agriculture: A Contingent Valuation Approach.” *Spanish Journal of Agricultural Research* 8(S2): 187. http://revistas.inia.es/index.php/sjar/article/view/1361 (May 9, 2019).

Barrio, Melina, and Maria Loureiro. 2013. “The Impact of Protest Responses in Choice Experiments: An Application to a Biosphere Reserve Management Program.” *Forest Systems* 22(1): 94–105. http://revistas.inia.es/index.php/fs/article/view/3103 (May 9, 2019).

Bateman, Ian J., Brett H. Day, Stavros Georgiou, and Iain Lake. 2006. “The Aggregation of Environmental Benefit Values: Welfare Measures, Distance Decay and Total WTP.” *Ecological Economics* 60(2): 450–60.

Birol, Ekin et al. 2013. “Using the Choice Experiment Method to Inform River Management in Poland: Flood Risk Reduction versus Habitat Conservation in the Upper Silesia Region.” In *Choice Experiments Informing Environmental Policy*, https://mpra.ub.uni-muenchen.de/id/eprint/38426 (May 9, 2019).

Bouscasse, Hélène et al. 2011. “Evaluation Économique Des Services Rendus Par Les Zones Humides - Enseignements Méthodologiques de Monétarisation.” : 220. http://www.developpement-durable.gouv.fr/IMG/pdf/ED49.pdf.

Brouwer, Roy, and Ian J. Bateman. 2005. “Temporal Stability and Transferability of Models of Willingness to Pay for Flood Control and Wetland Conservation.” *Water Resources Research* 41(3): 1–6. http://doi.wiley.com/10.1029/2004WR003466 (May 9, 2019).

Castro, Antonio J. et al. 2016. “Willingness to Pay for Ecosystem Services among Stakeholder Groups in a South-Central U.S. Watershed with Regional Conflict.” *Journal of Water Resources Planning and Management* 142(9): 05016006. http://ascelibrary.org/doi/10.1061/%28ASCE%29WR.1943-5452.0000671 (May 9, 2019).

Doherty, Edel, Geraldine Murphy, Stephen Hynes, and Cathal Buckley. 2014. “Valuing Ecosystem Services across Water Bodies: Results from a Discrete Choice Experiment.” *Ecosystem Services* 7: 89–97. https://www.sciencedirect.com/science/article/pii/S2212041613000624 (March 19, 2019).

Environment Agency. 2015. “Information on Selected Programmes of Measures for the 2nd Management Cycle.” https://s3-eu-west-1.amazonaws.com/data.defra.gov.uk/WaterQuality/wfd/Measures_Information.zip%0A (April 10, 2019).

Genius, M et al. 2008. “Evaluating Consumers’ Willingness to Pay for Improved Potable Water Quality and Quantity.” *Water Resources Management* 22(12): 1825–34. https://idp.springer.com/authorize/casa?redirect_uri=https://link.springer.com/article/10.1007/s11269-008-9255-7&casa_token=tr21PV_17NkAAAAA:WdPggBThHtKNaTlyyYC1ZMsU5U0rvAs_rOO88LHJ_ElcBiIG_zIwfd9v-in5YX_l_iF2QrjvgejQ4qM (May 9, 2019).

Genius, Margarita, Angeliki N. Menegaki, and Konstantinos P. Tsagarakis. 2012. “Assessing Preferences for Wastewater Treatment in a Rural Area Using Choice Experiments.” *Water Resources Research* 48(4). http://doi.wiley.com/10.1029/2011WR010727 (July 8, 2019).

He, Jie, Jérôme Dupras, and Thomas G. Poder. 2017. “The Value of Wetlands in Quebec: A Comparison between Contingent Valuation and Choice Experiment.” *Journal of Environmental Economics and Policy* 6(1): 51–78. https://www.tandfonline.com/doi/full/10.1080/21606544.2016.1199976 (May 7, 2019).

Hein, Lars. 2011. “Economic Benefits Generated by Protected Areas: The Case of the Hoge Veluwe Forest, the Netherlands.” *Ecology and Society* 16(2).

Koundouri, P., R. Scarpa, and M. Stithou. 2014. “A Choice Experiment for the Estimation of the Economic Value of the River Ecosystem: Management Policies for Sustaining NATURA (2000) Species and the Coastal Environment.” In *Global Issues in Water Policy*, , 101–12. http://link.springer.com/10.1007/978-94-007-7636-4_6 (July 8, 2019).

Markantonis, V, V Meyer, and N Lienhoop. 2013. “Evaluation of the Environmental Impacts of Extreme Floods in the Evros River Basin Using Contingent Valuation Method.” *Natural hazards*. http://link.springer.com/article/10.1007/s11069-013-0762-3 (January 5, 2017).

Polyzou, E, N Jones, K. I. Evangelinos, and C. P. Halvadakis. 2011. “Willingness to Pay for Drinking Water Quality Improvement and the Influence of Social Capital.” *Journal of Socio-Economics* 40(1): 74–80. https://www.sciencedirect.com/science/article/pii/S1053535710000831 (May 9, 2019).

Stithou, Mavra, Stephen Hynes, Nick Hanley, and Danny Campbell. 2012. “Estimating the Value of Achieving ‘Good Ecological Status’ in the Boyne River Catchment in Ireland Using Choice Experiments.” *Economic and Social Review* 43(3): 397–422. https://www.esr.ie/article/view/44 (May 9, 2019).
